# Pathophysiological model of chronic heart failure complicated with renal failure caused by three-quarter nephrectomy and subcutaneous injection of isoprenaline

**DOI:** 10.3892/etm.2012.865

**Published:** 2012-12-19

**Authors:** DING-FENG PENG, SHAO-YONG TANG, YONG-JUN HU, JIAO CHEN, LI YANG

**Affiliations:** Department of Vasculocardiology, Wuhan Puai Hospital, Wuhan 430033, P.R. China

**Keywords:** isoprenaline, heart failure, renal failure, Sprague-Dawley rats

## Abstract

This study aimed to investigate the pathophysiological changes in a rat chronic heart failure complicated with renal failure model, caused by three-quarters nephrectomy and subcutaneous injection of isoproterenol (ISO). Sprague-Dawley (SD) rats in the model group received three-quarters nephrectomy after twice undergoing surgical resections and subcutaneous injection of ISO (100 mg/kg body weight, injected twice, with a 24 h interval) after one week, while rats in the control group received sham surgery and injection of normal saline. Survival rate, heart failure and renal failure were compared between the two groups after 4 weeks. Serum creatinine (Cr), blood urea nitrogen (BUN), B-type natriuretic protein (BNP), aldolase (ALD), angiotensin II (Ang II) and C-reactive protein (CRP) were determined by kit assay. Urine protein at 24 h was determined by the Bradford method and left ventricular systolic pressure (LVSP), left ventricular diastolic pressure (LVDP) and left ventricular end-diastolic pressure (LVEDP), as well as the maximum rates of increased and decreased left ventricular pressure (±dP/dt_max_) were determined by left ventricular intubation. Heart weight indices were determined and the myocardial pathological conditions were observed by hematoxylin and eosin (HE) staining. There was no death in the control group, while the survival rate of the model group was 73%. Compared with the control group, each index of serum and urine protein in the model group was significantly increased. Additionally, LVSP was decreased, LVDP and LVEDP were increased and heart weight index was increased, with a significant difference. The serum Cr was positively correlated to BNP levels in the model group. Three-quarters nephrectomy and subcutaneous injection of ISO induces left ventricular heart failure and renal failure at the same time, which is characterized in pathophysiology by left ventricular diastolic and systolic function failure, left ventricular myocardial hypertrophy and reconstruction complicated with renal insufficiency.

## Introduction

Cardiorenal syndrome (CRS) is a complex problem. Patients complicated with heart failure and chronic renal insufficiencies are at a greater risk of poor prognosis. The incidences of heart failure and renal failure are gradually increasing, with poorer prognosis. CRS requires greater attention and research to develop therapeutic measures in reference to the fundamental mechanism of this disease. Currently, there are no animal models for studying heart and renal dysfunction. Therefore, it is necessary to establish an effective animal model of heart-kidney interaction in order to increase our understanding of this syndrome.

At present, there are a number of methods for inducing heart (1,2) and renal insufficiency (3), which theoretically could be united to establish a new disease model of heart-kidney interaction. However, none of these methods completely represent the pathogenesis and clinical features of human CRS. Therefore, an improved animal model of heart-kidney interaction is required. The pathological changes in an isoproterenol (ISO)-induced congestive heart failure (CHF) model in Sprague-Dawley (SD) rats are characterized by repeated and multiple focal myocardial necrosis, which is similar to ischemic heart disease. There have been reports on varying dosages for creating an ISO-induced CHF model in SD rats (4), in which subcutaneous injection of >85 mg/kg ISO is considered a large dose. The large dose has a high success rate of modeling, but with high mortality, while a small dose has a low success rate of modeling, but with greater longevity. The two models are detrimental to drug observation and research. The most common method used to induce renal failure reduces the survival of kidney tissue by different means, resulting in varying extents of removal and severity of renal insufficiency. Unilateral nephrectomy causes slight renal impairment, but without significant urine protein increases and histological changes (5). Renal sub-radical resection leads to more serious renal insufficiency, as well as uremia and complications of chronic kidney disease similar to humans (6,7). It has also been reported that renal sub-radical resection causes structural changes to the heart tissues (8–10). This study aimed to explore a new model of CRS and its pathological mechanism.

## Materials and methods

### Animals

Thirty SD male rats, weighing 180±20 g, provided by the Laboratory Animal Center of Tongji Medical College of Huazhong University of Science and Technology (China) were used in this study. This study was carried out in strict accordance with the recommendations in the Guide for the Care and Use of Laboratory Animals of the National Institutes of Health. The animal use protocol has been reviewed and approved by the Institutional Animal Care and Use Committee (IACUC) of Wuhan Puai Hospital.

### Modeling

Thirty SD male rats were randomly divided into a control group (n=15) and model group (n=15). After feeding for one week, the rats in the model group were narcotized with 10% chloral hydrate (0.3 ml/100 g intraperitoneal injection). The rats then received resection of the lower pole of the left kidney in the first week and radical resection of the right kidney after one week according to the previously described protocol of twice undergoing surgical resections (11). Rats in the control group underwent surgery twice, although not kidney resection. There was no death in the two groups after conventional feeding for one week. Subcutaneous injections of ISO (100 mg/kg body weight, injected twice) with an interval of 24 h were administered to the rats in the model group, while rats in the control group received two subcutaneous injections of normal saline (100 mg/kg body weight) with an interval of 24 h. All 30 rats received normal water and food for 4 weeks. The general conditions, including diet, hair color, activity, cyanosis and edema were observed and rats were weighed weekly.

### Protein determination

After 4 weeks, the rats in the two groups were placed into metabolism cages and urine was collected at 24 h. The Bradford method (Coomassie brilliant blue G-250 staining) was used to detect protein content in the urine and calculate the protein excretion of urine at 24 h. Intraocular venous blood was then collected to prepare serum and the levels of serum creatinine (Cr), blood urea nitrogen (BUN), B-type natriuretic protein (BNP), aldolase (ALD), angiotensin II (Ang II) and C-reactive protein (CRP) was detected.

### Hemodynamics

Approximately 24 h after blood collection, left ventricular intubation was administered via the left common carotid artery for left ventricular cardiac function testing. A neck incision was made to expose and bluntly separate the right common carotid artery. The left ventricular catheter was pre-charged with 10% heparin saline and was intubated against the right common carotid artery into the left ventricle. The other end of the catheter was connected to a pressure transducer (BL-420F biological function experimental system) (Chengdu Technology & Market Co. Ltd., Chengdu, China) for hemodynamic detection of left ventricular end-diastolic pressure (LVEDP) and left ventricular systolic pressure (LVSP), as well as the maximum rates of increased and decreased left ventricular pressure (±dP/dt_max_). Changes in cardiac function were calculated.

### Hematoxylin and eosin (HE) staining

The left ventricular tissues were fixed in 10X PBS or 4% neutral formaldehyde. The tissues paraffin-embedded and sliced to produce 4 *μ*m sections. The sections underwent HE staining and then images were captured with a Leica microscope (magnification, ×100).

### Statistical analysis

Data were expressed as the mean ± standard deviation. Comparison of the mean values between the groups was examined with the χ^2^ test and variance analysis. Data were analyzed with statistical software SPSS 11.5 (SPSS Inc., Chicago, IL, USA). P<0.05 was considered to indicate a statistically significant difference.

## Results

### General information

Compared with the control group, rats in the model exhibited darker hair, reduced feeding, decreased early weight, gradually increased later weight, slow movement, cyanosis in the mouth and nose, asthma, edema in the feet and paws, reduced activity and a poor grab reaction. In the model group, 3 rats died within 1 week and 4 rats died 4 weeks following the subcutaneous injections of ISO, administered twice, with a survival rate of 73%. No death was noted in the control group.

### Cardiac and renal function parameters

**Figure 1.**
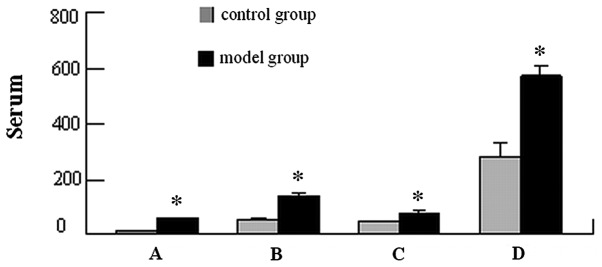
Comparison of BNP, ALD and Ang II between the control and model groups. ^*^P<0.01, compared with the control group. (A) Serum BNP (pg/ml); (B) ALD (*μ*g/ml); (C) Ang II (*μ*g/ml) and (D) CRP (*μ*g/ml). BNP, B-type natriuretic protein; ALD, aldolase; Ang II, angiotensin II; CRP, C-reactive protein.

**Figure 2.**
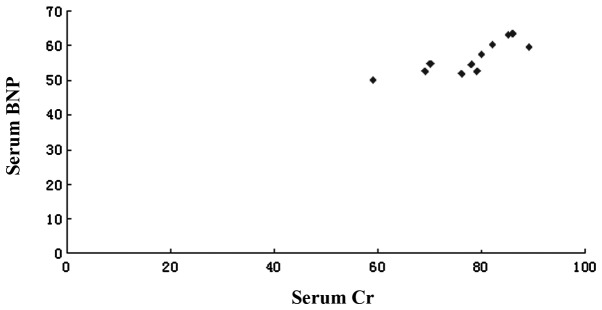
Correlogram of serum BNP and Cr. Serum BNP was positively correlated with serum Cr, with a correlation coefficient r=0.81, P<0.01. BNP, B-type natriuretic protein; Cr, creatinine.

Compared with the control group, serum Cr, BUN and urine protein in the model group were increased (P<0.01), which indicates a successful modeling of renal failure (Table I). All the rats in the model group demonstrated cardiac insufficiency, characterized by significantly decreased LVSP, increased LVDP and LVEDP and decreased dP/dt_max_ (P<0.05; Table II). Compared with BNP (13.77±2.38 pg/ml), ALD (51.57±9.17 *μ*g/l), Ang II (43.36±4.63 *μ*g/l) and CRP (282.9±47.58 *μ*g/l) in the control group, the serum BNP (56.48±4.67 pg/ml), ALD (137.69±16.13 *μ*g/l), Ang II (81.76±5.78 *μ*g/l) and CRP (568.54±42.15 *μ*g/l) were significantly increased in the model group (P<0.05; Fig. 1). Serum Cr was positively correlated with blood serum BNP levels in the model group (Fig. 2).

### Weight index

Compared with the control group, the weights of the rats in the model group were significantly reduced, whereas weights of the left ventricule and ventricular weight indices were significantly increased in the model group (P<0.01). A significant difference was detected between the weight of the left ventricle and the left ventricular weight index (P<0.01; Table III). Cardiac function indices demonstrated that the left ventricle suffered clear hypertrophy and reconstruction and entered the decompensatory stage of heart failure in the model group.

### HE staining

**Figure 3.**
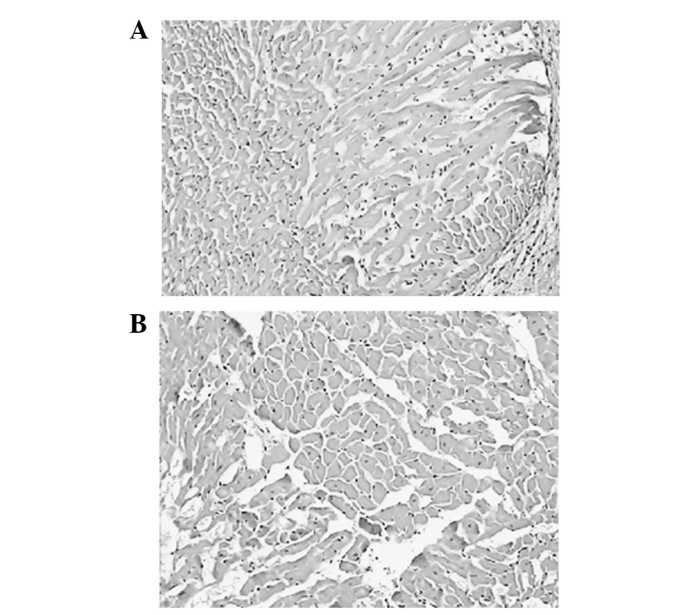
Hematoxylin and eosin (HE) staining of cardiac muscular tissue in the left ventricle. (A) Control group and (B) model group (magnification, ×100).

Following HE staining, slices were examined and compared with the control group. There was clear cardiomyocyte hypertrophy in the model group, with coarsened myocardial cells in an uneven arrangement and myocardial fibrosis. In the control group, myocardial cells were in even distribution, with fewer and inconspicuous extracellular matrices (Fig. 3).

## Discussion

The incidences of heart failure and renal failure has gradually increased. Renal insufficiency is an independent predictor of heart failure, while myocardial hypertrophy and heart function failure are serious complications of chronic renal failure, closely associated with mortality (12–14). In this study, a rat model of CRS was established to examine the pathophysiological mechanism in order to identify effective drugs and early intervention therapy.

A successful model of CRS, not only simulates the clinical characteristics of CRS, including changes in the heart, kidney, hemodynamics and neuroendocrinology, but also evaluates the therapeutic effect. This model must include joint damages of kidney and heart, characterized by progressive deterioration of cardio-nephric function. The model must present systolic dysfunction confirmed by echocardiography and haemodynamics, resulting in decreased cardiac output. In addition, to further explore the latest clinical findings, increased enddiastolic pressure and venous congestion are also necessary conditions. At a histological level, the model must present characteristics of hypertrophy and fibrosis, particularly mismatched myocardial/capillary. To successfully represent kidney damage, the model must present characteristics of increased Cr and excretion of albumin, as well as decreased progressive renal function caused by a decrease in the glomerular filtration rate (GFR)/Cr clearance ratio. This study aimed to explore the physiopathological mechanism of a CRS model induced by three-quarters nephrectomy and subcutaneous injection of ISO.

Compared with previous models of simple heart or renal failure, rats in this study presented earlier renal and heart failure, characterized by significantly increased Cr, urine protein and left ventricular weight index, as well as decreased hemodynamic index, including ±dP/dt_max_ and increased serum BNP. HE staining of the myocardium revealed clear hypertrophy of myocardial cells and myocardial fibrosis in the model group, indicating that reconstruction of myocardial hypertrophy had begun. Compared with heart failure caused only by ISO, the amount of subcutaneous injection of ISO was reduced in this model group. In the report of a CRS model by Van Dokkum *et al* and Windt *et al*(5,7), there was reduced interactive influence between heart and renal function. Dikow *et al*(6) hypothesized that the increase in Cr aggravated the left ventricular remodeling of myocardial infarction. We consider that the differences in experimental design may be associated with the varying degrees of damage to the heart and kidney, as well as different induction times. We analyzed indices of heart and kidney function in this model and identified that serum BNP was positively correlated to Cr in the model group, with a correlation coefficient of 0.81 (P<0.01), which is consistent with the study by Butler *et al*(15) and the retrospective study by Weinfeld *et al*(16). The hemodynamic variable associated with worsening renal function was right atrial pressure. Heart and renal function may influence each other in the occurrence and development of diseases. This model simulates the process of heart failure complicated with renal failure.

The possible mechanisms of heart-kidney interactions that have previously been considered include hemodynamic changes, endothelial dysfunction, inflammation, activation of the renin-angiotensin aldosterone system (RAAS) and/or the sympathetic system, any of which may cause cascade reactions of other factors, leading to structural and functional damage to the heart and kidney (17,18). The mechanism that causes and maintains heart-kidney interactions remains unclear. In this study, serum ALD and Ang II were significantly increased in the model group compared with the control group, indicating that activation of the RAAS is important in the occurrence of this model. At the same time, serum CRP was significantly increased in the model group compared with the control group (P<0.01), indicating that inflammation also plays a promoting role in this model of CRS. We also detected a marked change in urine protein, which is a strong and independent risk factor for cardiovascular disease. When it appears, proteinuria causes an accelerated atherosclerotic process, endothelial dysfunction, increased risk of terminal organ damage, serious cardiovascular events and mortality (19). Rats in this model suffer heart-kidney interactions, then when CRS occurs, the prognosis becomes significantly worse.

Due to the physiological changes of decreased renal function caused by three-quarters nephrectomy, this model had an increased sensitivity to adverse factors in chronic heart failure. Inflammation and activation of the RAAS promotes the occurrence and development of cardiorenal failure. Hemodynamic changes cause an increase in Ang II release, vasoconstriction, contraction of the efferent glomerular arteriole, cardiac remodeling and an increase in aldosterone release, as well as water and sodium retention, which promote myocardial fibrosis (20).

This study demonstrated that three-quarters nephrectomy complicated with subcutaneous injection of ISO induces concurrent heart and renal failure, with a high success rate, providing a simple, reliable and easy animal model for clinical discussion of the interactive mechanism of heart and renal function. However, this study did not investigate the development time of CRS, which is required to examine the illness and possible preventive therapeutic measures. This model is likely to be useful for further studies on the pathogenesis and development of heart-renal interaction at different stages.

## Figures and Tables

**Table I.** Comparison of urine protein, urea and creatinine between the control and model groups.

Group	Serum creatinine (*μ*mol/l)	Urea nitrogen (mmol/l)	Urine protein (mg/24 h)
Control	40.6±10.8	5.89±2.26	15.6±5.36
Model	77.5±8.7^a^	10.2±1.5^a^	70.5±12.7^a^

a P<0.01 vs. the control group. Data are presented as the mean ± standard deviation.

**Table II.** Comparison of left ventricular cardiac function and blood dynamic parameters between the control and model groups.

Group	LVSP (mmHg)	LVDP (mmHg)	LVEDP (mmHg)	+dP/dt_max_ (mmHg/sec)	−dP/dt_max_ (mmHg/sec)
Control	136.7±8.2	0.8±1.1	2.0±1.8	8060±892	−6902±949
Model	110.9±8.7^b^	1.8±0.6^a^	4.6±1.0^b^	5536±439^b^	−4036±413

a P<0.05 and

b P<0.01 vs. the control group. Data are presented as the mean ± standard deviation. LVSP, left ventricular systolic pressure; LVDP, left ventricular diastolic pressure; LVEDP, left ventricular end-diastolic pressure; ±dP/dt_max_, maximum rates of increased and decreased left ventricular pressure.

**Table III.** Comparison of weight, left ventricular mass and left ventricular mass index between the control and model groups.

Group	LVW (g)	BW (kg)	LVW/BW (g/kg)
Control	0.40±0.04	0.29±0.01	1.38±0.14
Model	0.57±0.04^a^	0.25±0.01^a^	2.29±0.19^a^

a P<0.01 vs. the control group. Data are presented as the mean ± standard deviation. LVW, left ventricular weight; BW, body weight.
